# Effects of size and microclimate on whole-tree water use and hydraulic regulation in *Schima superba* trees

**DOI:** 10.7717/peerj.5164

**Published:** 2018-07-06

**Authors:** Xiao-wei Zhao, Lei Ouyang, Ping Zhao, Chun-fang Zhang

**Affiliations:** 1 College of Life Sciences, Yulin University, Yulin, Shan Xi, PR China; 2 Key Laboratory of Vegetation Restoration and Management of Degraded Ecosystems, Guangdong Provincial Key Laboratory of Applied Botany, South China Botanical Garden, Chinese Academy of Sciences, Guangzhou, Guangdong, PR China; 3 Department of Life Sciences, Yuncheng College, Yuncheng, Shan Xi, PR China

**Keywords:** Sap flux density, Transpiration, Leaf water potential, Hydraulic conductance, Canopy conductance

## Abstract

**Background:**

Plant-water relations have been of significant concern in forestry and ecology studies in recent years, yet studies investigating the annual differences in the characteristics of inter-class water consumption in trees are scarce.

**Methods:**

We classified 15 trees from a *Schima superba* plantation in subtropical South China into four ranks using diameter at breast height (DBH). The inter-class and whole-tree water use were compared based on three parameters: sap flux density, whole-tree transpiration and canopy transpiration over two years. Inter-class hydraulic parameters, such as leaf water potential, stomatal conductance, hydraulic conductance, and canopy conductance were also compared.

**Results:**

(1) Mean water consumption of the plantation was 287.6 mm over a year, 165.9 mm in the wet season, and 121.7 mm in the dry season. Annual mean daily water use was 0.79 mm d^−1^, with a maximum of 1.39 mm d^−1^. (2) Isohydrodynamic behavior were found in *S. superba*. (3) Transpiration was regulated via both hydraulic conductance and stoma; however, there was an annual difference in which predominantly regulated transpiration.

**Discussion:**

This study quantified annual and seasonal water use of a *S. superba* plantation and revealed the coordinated effect of stoma and hydraulic conductance on transpiration. These results provide information for large-scale afforestation and future water management.

## Introduction

Plantation species that are artificially planted in rows in intensively managed stands have different ecological functions to that of natural forests; in particular their water use is significantly higher (Farley, Jobbagy & Jackson, 2005; Nosetto, Jobbágy & Paruelo, 2005; Licata et al., 2008). As sap flow technologies have become widespread, studies of whole-tree water relations have increased rapidly. Many of these studies focus on water consumption of planted trees, because of the potential risk to the water balance over a large area (Morris et al., 2004; Little et al., 2009; Rascher et al., 2011). However, these studies focus more on inter-species than within-species comparisons.

China is the second largest plantation country in the world and has established extensive areas of plantations since the 1950s. The planting programs during the 1970s and 1980s mainly focused on fast-growing, high-yielding timber species, as well as ecosystem protection and rehabilitation (FAO, 2010; FSC, 2012). The rapid and large-scale simultaneous expansion of the size and growth of plantations caused concern in both researchers and the public over the balance of ground water, which was mainly concentrated in southern China and the Yellow River basin. Studies of plantation size focused on productive species containing fast-growing, economically valuable species (Lane et al., 2004; Ma et al., 2008; Tan et al., 2011; Zhu et al., 2015), while studies of plantation growth focused more on species of ecological recovery (Ge et al., 2006; Cao et al., 2007; Cao, Chen & Yu, 2009; Chen et al., 2010; Wang et al., 2010; Zheng et al., 2012; Jian et al., 2015). These studies drew different conclusions: that pure plantations: (1) posed a potential risk to ground water and were even considered as water pumps; (2) had a higher potential to reduce soil water storage in the watershed; and (3) posed no risk to ground water, but had potential hydrologic responses to climate and soil conditions. Therefore, more stands need to be studied.

In the Guangdong Province of China, afforestation efforts have increased the forest cover from approximately 20% in the 1950s to approximately 60% at present (Zhou et al., 2008). *Schima superba* is a native tree species in south China, and plantations cover up to 1,236 hm^2^ in Guangdong Province (Hu et al., 2007). In 2008, a study on *S. superba* water relations was conducted in the South China Botanical Garden. Previous studies compared seasonal hydraulic characters of *S. superba,* including sap flux density, whole-tree transpiration, and hydraulic conductance (Mei et al., 2010a; Zhu et al., 2010; Zhang et al., 2014; Zhou et al., 2015). However, at the individual-scale, we lack an understanding of drought stress strategy, and coordinated regulation of transpiration via stoma and hydraulic conductance. At the stand-scale, we have not yet quantified the effects of rainfall on annual and seasonal patterns of *S. superba* water use, or even the annual consumption of water.

As one of the major determinants of the rate of water flux through trees at the stand-scale, many studies have evaluated *g*_*c*_, mainly using the JS modeling approach (Martin et al., 1997; Magnani et al., 1998; Zhang, He & Assmann, 2008) and PM equations ((Morris, Mann & Collopy, 1998; Granier, Biron & Lemoine, 2000a; Harris et al., 2004); Komatsu et al. 2006; Whitley et al., 2009). However, less work was done in comparing inter-class canopy conductance.

In this study, our objectives were to: (1) quantify water use for each tree rank and the plantation as a whole; (2) determine iso/anisohydry; (3) quantify inter-class canopy conductance and verify the possibility of a mean instantaneous conductance substitute for canopy conductance in the same canopy layer; and (4) determine if stomatal conductance determines water use more than hydraulic conductance.

## Materials & Methods

### Site description

The experiments were conducted in a *S. superba* plantation (23°10′N, 113°21′E, 41.4 m alt) that was planted in the mid-1980s and located in the ecological observation station of the South China Botanical Garden, Chinese Academy of Sciences, Guangzhou. The study area had a subtropical monsoon climate, with an annual mean temperature of 21.8 °C, and mean temperature of 32.7  °C in the hottest month of July, and 9.8 °C in the coldest month of January. Annual mean rainfall was 1750 mm, with April to September tending to be wet, and October to March of the next year tending to be dry. In 2010, the annual mean rainfall was 2148.4 mm and in 2011, annual mean rainfall was 1421.2 mm (Guangzhou Bureau of Statistics, 2010; Guangzhou Bureau of Statistics, 2011). The soil was loamed with an organic content of 2.3% and total nitrogen content of 0.07%. This *S. superba* plantation was planted at a density of 603 plants/hm^2^, and the mean height of the stand at the time of the study was 12.7 m. The experimental site covered approximately 2885.6 m^2^.

**Table 1:** Tree form features of 21 sampled trees. All trees were measured in April, 2011.

Tree No.	Diameter at breast height (*DBH*/m)	Height (m)	Canopy diameter (m^∗^m)	Sapwood area (*A*_*s*_∕m^2^)	Leaf area (*A*_*L*_∕m^2^)
1	0.15	15.30	6.4 × 2.3	0.016	66.97
2	0.19	12.60	6.7 × 4.3	0.025	101.83
3	0.13	12.10	4.5 × 2.3	0.012	54.16
4	0.22	15.30	6.6 × 5.6	0.031	125.66
5	0.22	15.50	6.9 × 5.3	0.032	129.50
6	0.10	11.00	1.2 × 0.9	0.007	30.86
7	0.18	12.90	5.5 × 5.0	0.020	85.71
8	0.09	9.70	3.4 × 3.9	0.006	27.15
9	0.09	9.50	2.3 × 2.6	0.006	27.15
10	0.24	16.90	7.0 × 6.2	0.036	145.34
11	0.14	11.20	2.9 × 4.3	0.013	55.53
12	0.07	8.00	2.0 × 2.6	0.004	16.36
13	0.08	12.00	3.1 × 1.8	0.006	25.12
14	0.14	13.10	4.4 × 3.1	0.014	61.86
15	0.07	9.70	2.4 × 1.8	0.004	19.86
16	0.19	13.70	4.6 × 4.8	0.025	101.83
17	0.21	15.40	7.8 × 4.6	0.029	118.11
18	0.20	14.90	5.4 × 5.8	0.026	108.04
19	0.14	11.20	4.1 × 4.9	0.014	59.72
20	0.15	13.40	4.4 × 4.5	0.015	64.76
21	0.26	13.90	6.4 × 6.8	0.041	164.03

### Tree architecture characteristics

Characteristics of the sampled trees were measured in April 2011 to avoid age effects on transpiration (Table 1) (Zhang et al., 2014). Trees 1–15 were used to estimate transpiration and were divided into four groups based on diameter at breast height (DBH) (Table 2) Trees 16–21 were used to measure leaf water potential and hydraulic conductance, and were classified into three groups according to DBH (Table 2). Trees 17–18 and 21 were used to analyze stomatal conductance. Tree height (H, m) was measured using a clinometer (Suunto Oy, Vantaa, Finland). DBH was measured using a diameter tape. Cores from the stems of five trees outside and close to the plot were taken using an increment core borer. The sapwood depth was measured using a caliper as sapwood and heartwood in *S. superba* are easy to identify visually. An exponential regression (see Eqs. (3-1), (2) and (3) below) between the sapwood area (*A*_*s*_, m^2^) and DBH was established based on the above measures, which was used to calculate the *A*_*s*_ of the 15 trees in the plot. Similarly, leaf area (*A*_*L*_) was calculated using a log function (see (6) below) established from the five trees outside and close to the plot.

**Table 2:** Features of plot and sample trees used in classification. DBH standed for diameter at breast height. Number of trees (I) showed tree numbers of stand. Number of trees (II) showed sampling tree numbers for evaluated stand water use. Number of trees (III) showed sampling tree numbers for measuring leaf water potential and instantaneous stomatal conductance (Tree No. 17 ∼18, 21).

	Total	Rank 1	Rank 2	Rank 3	Rank 4
		DBH > 0.20 m	0.15 m < DBH ⩽ 0.20 m	0.10 m < DBH ⩽ 0.15 m	0.05 m < DBH ⩽ 0.10 m
Plot (2,885.6m^2^)					
Number of trees (I)	174	14	48	71	41
Sample trees					
Number of trees (II)	15	No. 4, 5, 10	No.1, 2, 7	No.3, 11, 14	No. 6, 8, 9, 12, 13, 15
*A*_*s*_ (m^2^)	0.081	0.037	0.023	0.015	0.006
Projected canopy area	15.85	7.65	4.65	2.39	1.16
Number of trees (III)	6	No.17, 21	No.16, 18	No.19, 20	
*A*_*s*_ (m^2^)	0.028	0.040	0.028	0.016	

### Environmental measurements

We continuously monitored wind speed (*WS*, m s^−1^), air temperature (*TA*, °C), relative humidity (*RH*, %), and photosynthetically active radiation (*PAR*, µmol m^−2^ s^−1^) above the *S. superba* forest canopies from an observation tower set up within the plantation. Soil moisture (*SM*, m^−3^ m^−3^) was measured 30 cm below the ground surface from October 2010 until December 2011. Wind speed was measured using cup anemometers (AN4; Delta-T Devices, UK). *TA* and *RH* were measured using a temperature and humidity sensor (RHT2V-418; Delta-T Devices, UK). *PAR* was measured using a Li-Cor quantum sensor (LI-190SA; LI-COR, USA). *SM* was measured using three frequency domain sensors (SM200; Delta-T Devices) at a depth of 30–40 cm, which were set in a triangle around trees 17–21. The output values were measured every 30 s, and 10-min mean values were logged using a DL2e Delta-T logger (DL2e; Delta-T Devices).

The vapor pressure deficit (*VPD*, kPa) combined with the parameters *TA* and *RH* was calculated using the following formula (Campbell & Norman, 1998):

(1) \begin{eqnarray*}VPD={\mathrm{ae}}^{ \left( \frac{bTA}{TA+c} \right) }(1-RH)\end{eqnarray*}

Where, a, b, and c are fixed parameters (0.611 kPa, 17.502 [unitless], and 240.97 °C, respectively).

### Sap flux density measurements

Sap flux density (*J*_*s*_) was continuously measured from 2010 until 2011 using a homemade thermal dissipation probe (TDP). The Grainer-type probes were inserted radially at a depth of 20 mm into the stem of 21 trees at 1.3 m aboveground, one pair for each tree. The upper heated probe was supplied with a constant DC current (120 mA). The unheated probe was installed 10–15 cm below the upper sensors. The temperature difference between the paired sensors was recorded using the same logger as environmental measurements. All probes were installed on the north-facing side of the trees and covered with a plastic case, and shielded using a radiation-insulating film in order to minimize the possible effect of direct sunshine.

Sap flux density (*J*_*s*_, g m^−2^ s^−1^) was weighted using the following equation:

(2) \begin{eqnarray*}{J}_{s}=\lambda (40\ast {J}_{0-40}+ \left( d-40 \right) \ast {J}_{40})/d(d\gt 40)or{J}_{0-40}(d\leqslant 40)\end{eqnarray*}

where, *J*_0−40_ (Sap flux density at 0–40 mm) is calculated using the empirical equation proposed by Granier (1987), d is sapwood thickness, and *λ* are correction factors of 1.164, 1.128, and 1.096 for Ranks 1, and 3, respectively. *J*_40_ (sap flux density at ⩾40 mm) was calculated following Mei et al. (2010b):

(2-1) \begin{eqnarray*}{J}_{40}=0.45\times {J}_{0-40}\end{eqnarray*}

Whole-tree transpiration (*F*_*s*_, g d^−1^) was calculated as:

(3) \begin{eqnarray*}{F}_{s}=\sum ({J}_{0-40}\times {A}_{0-40}+{J}_{40}\times {A}_{40})\times t\end{eqnarray*}

where, *t* is 600 s (data were averaged and stored every 10 min in the logger), *A*_0−40_ and *A*_40_ are the sapwood areas in the outer xylem (0–40 mm) and inner xylem (⩾40 mm), respectively, which were calculated as:

(3-1) \begin{eqnarray*}{A}_{0-40}=0.166\bullet { \left( DBH \right) }^{1.336}\end{eqnarray*}

(3-2) \begin{eqnarray*}{A}_{40}={A}_{s}-{A}_{0-40}\end{eqnarray*}

where *A*_*s*_ is the total sapwood area (*m*^2^), calculated using the regression equation relating it to *DBH* (m) as follows:

(3-3) \begin{eqnarray*}{A}_{s}=0.465\bullet { \left( DBH \right) }^{1.794}.\end{eqnarray*}

Monthly water use (∑*F*_*s*_, g mon^−1^) was calculated as:

(4) \begin{eqnarray*}\sum {F}_{s}=30\times {F}_{s}\end{eqnarray*}

Monthly canopy transpiration (∑*E*_*L*_, g m^−2^ mon^−1^) was calculated as:

(5) \begin{eqnarray*}\sum {E}_{L}=30\times {E}_{L}=30\times \frac{{F}_{s}}{{A}_{L}} \end{eqnarray*}

Where, *E*_*L*_ is canopy transpiration (g m^−2^ d^−1^), *A*_*L*_ is the total leaf area (m^2^), which was calculated using a regression equation relating it to DBH (m) as follows (Schäfer, Oren & Tenhunen, 2000):

(6) \begin{eqnarray*}\log \nolimits {A}_{L}=1.672\bullet \log \nolimits DBH+3.199\end{eqnarray*}

Annual stand transpiration (*E*_*s*_, mm y^−1^) was calculated from ∑∑*F*_*s*_ per ground area over 1 year. Δ*E*_*L*_ was the difference between predawn and midday *E*_*L*_

### Leaf water potential and stomatal conductance

The branches sampling procedure was conducted on trees 16–21 over 5–7 continuous sunny days in five time periods, Oct 2010, Jan, Apr, Jul, and Oct 2011. Leaf water potential (*ψ*_*L*_, MPa) was measured using a portable pressure chamber in two ways: at 1-hour intervals from 05:00–06:00 to 20:00 in Oct 2010, and point measuring at 05:00–06:00, 13:00, and 20:00 in the other time periods. This minimized the destructive sampling of tree branches. Predawn leaf water potential (*ψ*_*pd*_, MPa) and midday leaf water potential (*ψ*_*md*_, MPa) referred to *ψ*_*L*_ at 05:00–06:00 and 13:00, respectively. Iso/anisohydraulic regimes during the time periods were determined using the linear framework of (Martinez-Vilalta et al., 2014):

(7) \begin{eqnarray*}{\psi }_{md}=\Lambda +\sigma {\bullet \psi }_{pd}\end{eqnarray*}

Where Λ is the intercept of the relationship, and *σ* is the slope. A *σ* = 0 implies strict isohydry, *σ* = 1 implies strict anisohydry, *σ*1 implies extreme anisohydry and 0 < *σ* < 1 implies partial isohydry.

Instantaneous stomatal conductance (*g*_*s*_, m s^−1^) was simultaneously measured on trees 17–18 and 21 using a Li-6400 Portable Photosynthesis System, at the same time intervals and periods as *ψ*_*L*_.Δ*g*_*s*_ represented the difference between predawn and midday *g*_*s*_. All measurements were taken three times.

### Canopy conductance and whole-tree hydraulic conductance

Canopy conductance (*g*_*c*_, m s^−1^) was calculated by inverting the following equation (Lu et al., 2003):

(8) \begin{eqnarray*}\lambda {E}_{c}= \frac{\delta {R}_{n}+{K}_{t}\rho {C}_{p}\mathrm{V PD}{g}_{a}}{\lambda [\delta +\gamma (1+{g}_{a}/{g}_{c})]} \end{eqnarray*}

where, *λ* is the latent heat of vaporization (2.39 MJ kg^−1^), *E*_*c*_ (mm h^−1^) is estimated from whole-tree transpiration divided by the projected canopy area of 3.96 m^2^, *δ* is the slope of the relationship between saturated vapor pressure and temperature (kPa °C^−1^), *R*_*n*_ isthe net radiation above the forest canopy (MJ m^−2^ h^−1^), *K*_*t*_ is a unit conversion equal to 3,600 s h^−1^, *ρ* is air density (1.29 kg m^−3^), *C*_*p*_ is the specific heat of air (1.013 MJ kg^−1^ °C ^−1^), *γ* is the psychometric constant (0.066 kPa °C^−1^), and *g*_*a*_ isaerodynamic conductance (m s^−1^), which was calculated using the method in Delzon et al. (2004).

Whole-tree sapwood-specific hydraulic conductance *K*_*p*_ and leaf-specific hydraulic conductance *K*_*L*_ (g m^−2^ s^−1^ Mpa^−1^) were indirectly estimated following Cochard, Bréda & Granier (1996):

(9) \begin{eqnarray*}{K}_{p}={J}_{d}/({\psi }_{pd}-{\psi }_{md}) \text{or}{K}_{L}={J}_{l}/({\psi }_{pd}-{\psi }_{md})\end{eqnarray*}

where, *J*_*d*_ and *J*_*l*_ are the difference in sap flux density of sapwood and leaf area between predawn and midday (g m^−2^ s^−1^), respectively. We ignored time lags between sap flux density and transpiration (Zhao et al., 2016).

### Data analysis

January and July represented the dry and wet seasons, respectively, and October 2010 and 2011 represented annual differences in south China, respectively. All statistical analyses were performed using SPSS 15.0 (SPSS, Chicago, IL, USA) and Origin 8.1 (OriginLab, Northampton, MA, USA). The paired-samples *t*-test was used to analyze the seasonal and annual differences in *K*_*p*_ and *K*_*L*_. Major factors affecting *E*_*L*_ and *F*_*s*_ were determined using multilinear regression. All data were standardized before modeling. The strengths of relations were evaluated using the absolute values of the coefficients of separate models.

## Results

### Effects of tree size on daily sap flux density, whole-tree and canopy transpiration

**Figure 1:**
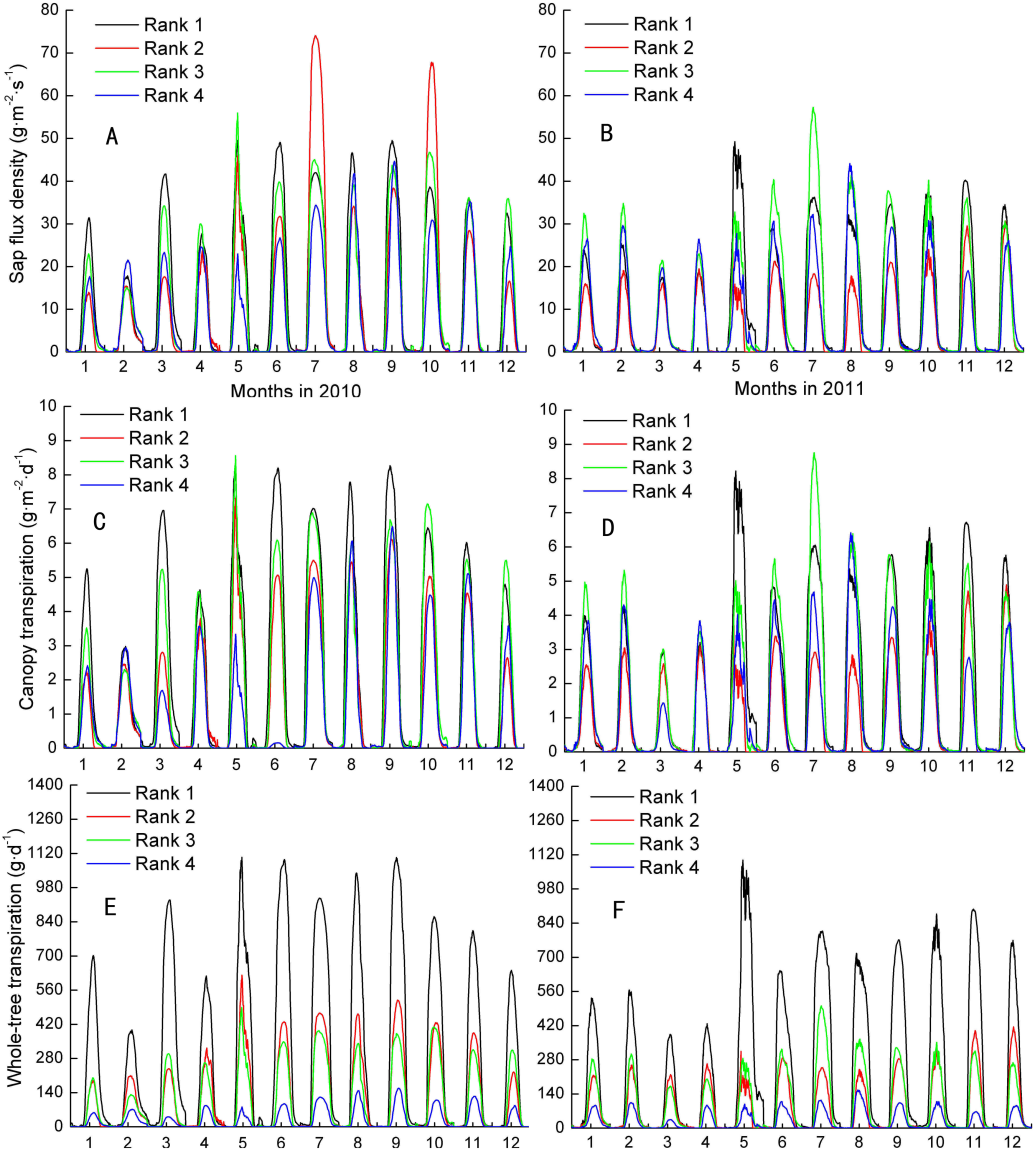
Inter-classes comparison in sap flux density, canopy transpiration and whole-tree transpiraiton. Rank 1 represented trees at DBH > 0.20 m; Rank 2 was trees at 0.15 m< DBH ≤ 0.20 m; Rank 3 was trees at 0.10 m< DBH ≤ 0.15 m; Rank 4 represented trees at 0.05 m< DBH≤ 0.10 m.

*J*_*s*_, *F*_*s*_, and *E*_*L*_ varied month by month, although all were at their maximums in the wet season and at their minimums in the dry season. The inter-class daily maximum of *J*_*s*_ followed weakly their size in 2010 and 2011 (Figs. 1A–1B). The total occurring rate of *J*_*s*_ ranking of Ranks 1 and 4 mapping their size ranking were 33.3% and 25.0%, respectively, which were higher than 4.2% and 12.5% of trees ranked 2 and 3 during the 2 years. Moreover, the separate mapped rate for Ranks 1-4 in 2010 were higher than that in 2011 except Rank 2, i.e., 41.7%, 0%, 16.7% and 33.3% compared with 25.0%, 8.3%, 8.3% and 16.7%, in turn. The total and separate mapped rates on *E*_*L*_ for the four Ranks were similar to that of *J*_*s*_ (Figs. 1C–1D). In contrast, the mapped rates on *F*_*s*_ increased up to 100% and 100% in Ranks 1 and 4, respectively, and up to 58.3% and 58.3% in Ranks 2 and 3, respectively (Figs. 1E–1F). Meanwhile, the separate mapped rates for all the four Ranks in 2010 were no different from that in 2011. *F*_*s*_ was evidently more close to present the assumption of the larger the tree, the greater the transpiration among the three parameters.

### Seasonal patterns of rank water use and whole-tree transpiration

**Figure 2:**
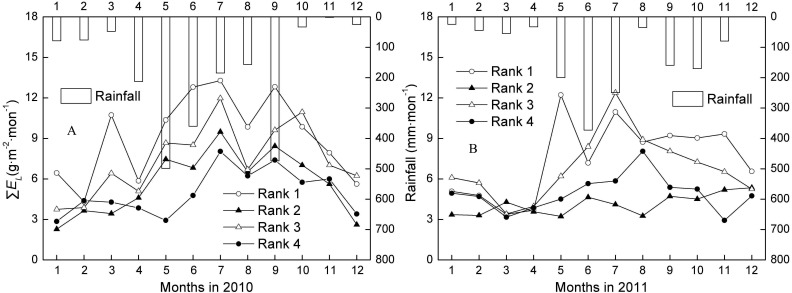
Changes of monthly canopy transpiration and rainfall in 2010 and 2011. Rank 1 represented trees at DBH > 0.20 m; Rank 2 was trees at 0.15 m < DBH ≤0.20 m; Rank 3 was trees at 0.10 m < DBH ≤0.15 m; Rank 4 represented trees at 0.05 m < DBH ≤0.10 m. ∑*E*_*L*_ was monthly canopy transpiration.

The ∑*F*_*s*_ and ∑*E*_*L*_ of trees ranked 1–4 generally showed a low-high-low trend, which followed the change in rainfall over the dry season (one to three months)-wet season (four to nine months)-dry season (10–12 months) (Figs. 2A–2B). The mean annual ∑*F*_*s*_ for Ranks 1, 2, 3, and 4 were 1301.28, 472.69, 469.28, and 135.34 g mon^−1^ in the wet season, and 924.25, 348.05, 345.53, and 106.65 g mon^−1^ in the dry season. The mean annual ∑*E*_*L*_ for Ranks 1, 2, 3, and 4 were 9.75, 5.57, 8.21, and 5.54 g m^−2^ mon^−1^ in the wet season, and 6.92, 4.10, 6.04, and 4.37 g m^−2^ mon^−1^ in the dry season. ∑*F*_*s*_ and ∑*E*_*L*_ of all tree ranks in the wet season were averagely 1.37 and 1.35 times higher than in the dry season during 2010 and 2011. That is, water use in the fast growth period was almost always more than 50%.

### Annual patterns of stand water use

The total water use for Ranks 1, 2, 3, and 4 were 312.8, 185.9, and 126.9 mm in the wet season, and 262.4, 145.9 and 116.5 mm in the dry season in 2010. The mean daily *E*_*s*_ was 0.86 mm d ^−1^ in 2010 and 0.72 mm d^−1^ in 2011. Moreover, *F*_*s*_ of all ranks declined to a certain extent, i.e., Ranks 1 and 2 declined from 40.2 and 15.8 g d^−1^ in 2010 to33.0 and 11.2 g d^−1^ in 2011, respectively, while Ranks 3 and 4 declined from 13.9 and 4.0 g d ^−1^ in 2010 to 12.9 and 3.9 g d^−1^ in 2011, respectively.

The stand ∑*F*_*s*_ was determined mainly by soil moisture (*SM*) and rainfall, with the effect of *SM* greater than that of rainfall (Table 3). Of the five factors (*PAR*, *VPD*, *SM*, *WS,* and rainfall), only *SM* was significant in the linear regression. Of the remaining four factors, rainfall was more significant than *WS* in the linear regression. Of *PAR*, *VPD*, and *WS*, no significant relations were observed. However, ∑*E*_*L*_ was determined mainly by *SM*, *PAR*, and rainfall, with the effect of *SM* >*PAR* >Rainfall >*WS* (Table 3). No relations were observed between ∑*E*_*L*_, *VPD*, and *WS*.

**Table 3:** Mutilinear regression analysis for *F*_*s*_, *E*_*L*_, Δ*E*_*L*_, *PAR*, *VPD*, *SM,WS*, Rainfall, *g*_*s*_ and *K*_*L*_.

Time	Depentent variable	*n*	Entered variables	Model	*R*^2^	*P*
2011	*F*_*s*_	15	*PAR*, *VPD*, *SM*, *WS*, Rainfall	*y* = 3.65 × 10^−16^ + 0.676∗*SM*	0.46	0.016
2011	*F*_*s*_	15	*PAR*, *VPD*, *WS*, Rainfall	*y* = − 2 × 10^−16^ + 0.573∗Rainfall − 0.515∗*WS*	0.60	0.016
2011	*F*_*s*_	15	*PAR*, *VPD*, Rainfall	*y* = − 9 × 10^−17^ + 0.579∗Rainfall	0.34	0.049
2011	*E*_*L*_	15	*PAR*, *VPD*, *SM*, *WS*, Rainfall	*y* = − 1 × 10^−17^ + 0.739∗*SM*	0.55	0.006
2011	*E*_*L*_	15	*PAR*, *VPD*, *WS*, Rainfall	*y* = − 6 × 10^−16^ + 0.512∗*PAR* + 0.498∗Rainfall	0.59	0.019
2011	*E*_*L*_	15	*PAR*, *VPD*, *WS*/*PAR*, *VPD*	*y* = − 6 × 10^−16^ + 0.588∗*PAR*	0.35	0.044
2011	*E*_*L*_	15	*VPD*, *WS*, Rainfall	*y* = − 6 × 10^−16^ + 0.571∗Rainfall − 0.54∗*WS*	0.62	0.012
Oct 2010	Δ*E*_*L*_	15	*K*_*L*_, Δ*g*_*s*_	*y* = − 2.04 × 10^−16^ + 0.874∗*g*_*s*_ − 0.282∗*K*_*L*_	0.77	0.000
Jan 2011	Δ*E*_*L*_	15	*K*_*L*_, Δ*g*_*s*_	*y* = − 1 × 10^−16^ − 0.021∗*g*_*s*_ − 0.678∗*K*_*L*_	0.47	0.023
Jul 2011	Δ*E*_*L*_	13	*K*_*L*_, Δ*g*_*s*_	*y* = 3.23 × 10^−16^ − 0.284∗*g*_*s*_ + 0.721∗*K*_*L*_	0.48	0.038
Oct 2011	Δ*E*_*L*_	15	*K*_*L*_, Δ*g*_*s*_	*y* = − 3 × 10^−17^ − 0.079∗*g*_*s*_ − 0.633∗*K*_*L*_	0.42	0.037

**Notes.**

*F*_*s*_ standed for whole-tree transpiration; *E*_*L*_ was canopy transpiration; Δ*E*_*L*_was the difference between predawn and midday *E*_*L*_; *PAR* standed for photosynthetically active radiation; *VPD* represented vapor pressure deficit; *SM* was soil moisture; *WS* was wind speed; *g*_*s*_ represented stomatal conductance; Δ*g*_*s*_ represented the difference between predawn and midday *g*_*s*_; *K*_*L*_ represented leaf-specific hydraulic conductance; *R*^2^ was the coefficient of determination; *p* was significance. *p* < 0.1, *p* < 0.05, and *p* < 0.01 standed for a significant, remarkable and very significant difference, respectively.

### Leaf water potential

**Figure 3:**
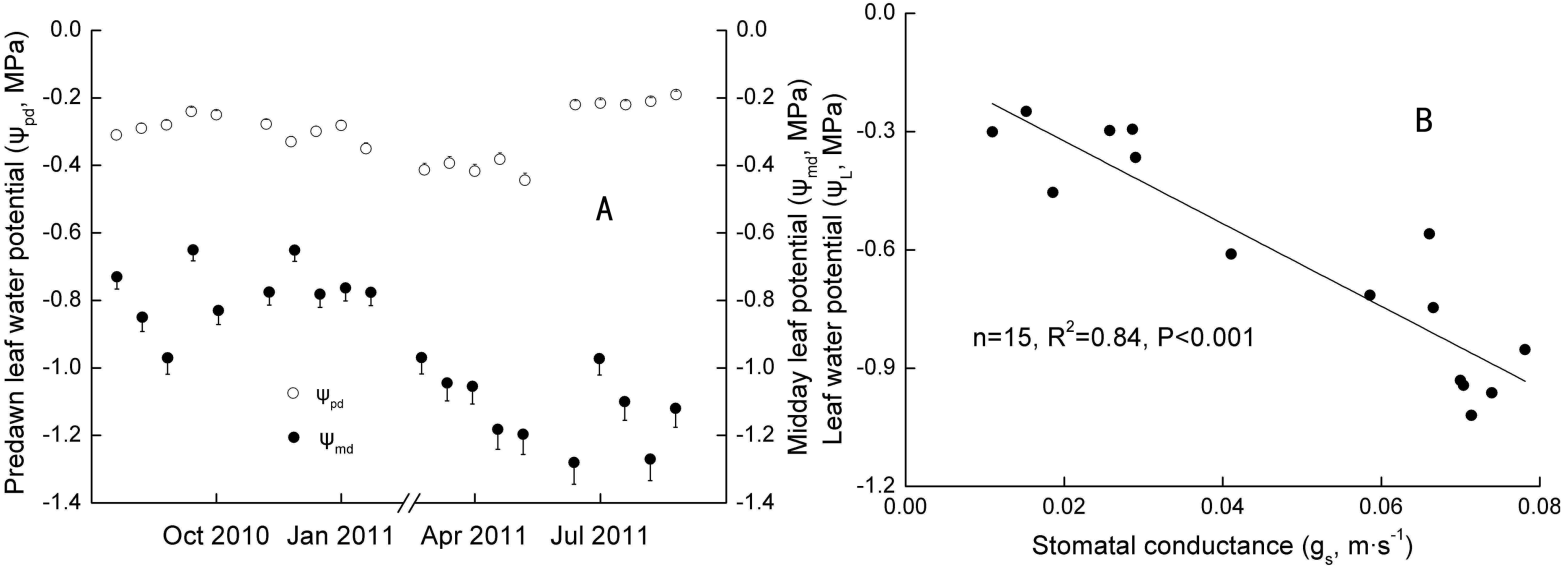
Seasonal changes and Linear regression of predawn and midday water potential. *R*^2^ was the coefficient of determination; *p* was significance. *p* < 0.1, *p* < 0.05, and *p* < 0.01 standed for a significant, remarkable and very significant difference, respectively.

Significant seasonal differences were observed in the *ψ*_*md*_ − *ψ*_*pd*_ for Ranks 1, 2 and 3 (Table 4). Daily *ψ*_*pd*_ and *ψ*_*md*_ ranged from −0.45 to −0.20 MPa and −0.97 to −0.61 MPa in Jan with *SM* at 23.39∼23.91 m^−3^ m^−3^, and −0.25 to −0.16 MPa and −1.47 to −0.68 MPa in Jul with *SM* at 34.57∼35.03 m^−3^ m^−3^ in 2011 (Fig. 3A). However, no significant annual differences were found in the *ψ*_*md*_ − *ψ*_*pd*_ for the three ranks (Table 4). Daily *ψ*_*pd*_ ranged from −0.42 to −0.18 MPa and −0.40 to −0.20 MPa, and the *ψ*_*md*_ ranged from −1.27 to −0.35 MPa and −1.33 to −0.56 MPa in the Oct 2010 and 2011, respectively.

**Table 4:** Paired samples *t* Test for *ψ*_*md*_ − *ψ*_*pd*_, *K*_*p*_ and *K*_*L*_. *ψ*_*md*_ was midday leaf water potential; *ψ*_*pd*_ was predawn water potential; *K*_*p*_ was sapwood-specific hydraulic conductance; *K*_*L*_ was leaf-specific hydraulic conductance. *SD* was standard deviation; *df* was degrees of freedom; *p* was significance. *p* < 0.1, *p* < 0.05, and *p* < 0.01 represented a significant, remarkable and very significant difference, respectively.

Test	*n*	Annual difference							Seasonal difference						
		Oct 2010		Oct 2011		*t*	*df*	*p*	Jan 2010		Jul 2011		*t*	*df*	*p*
		Mean	SD	Mean	SD				Mean	SD	Mean	SD			
*ψ*_*md*_ − *ψ*_*pd*_ in Rank 1	10	−0.58	0.30	−0.62	0.27				−0.42	0.11	−1.09	0.17			
Result						−0.52	9	0.614					9.68	9	0.000
*ψ*_*md*_ − *ψ*_*pd*_ in Rank 2	10	−0.58	0.24	−0.58	0.14				−0.50	0.13	−0.98	0.22			
Result						−0.01	9	0.996					6.30	9	0.000
*ψ*_*md*_ − *ψ*_*pd*_ in Rank 3	10	−0.52	0.27	−0.52	0.14				−0.38	0.04	−0.76	0.21			
Result						−0.10	9	0.956					5.82	9	0.000
*K*_*p*_ in Rank 1	10	59.91	17.40	57.84	13.93				58.49	13.32	39.33	14.88			
Result						0.27	9	0.793					1.90	9	0.021
*K*_*p*_ in Rank 2	10	70.69	23.40	44.37	10.84				83.75	31.84	23.40	8.67			
Result						2.66	9	0.026					4.65	9	0.000
*K*_*p*_ in Rank 3	10	86.57	53.04	37.00	15.17				73.58	43.71	62.77	25.85			
Result						3.70	9	0.005					0.23	9	0.614
*K*_*L*_ in Rank 1	10	1.67	0.48	1.62	0.40				1.64	0.37	3.36	1.26			
Result						0.25	9	0.811					−4.28	9	0.002
*K*_*L*_ in Rank 2	10	1.92	0.64	1.20	0.29				2.28	0.87	1.34	0.71			
Result						2.66	9	0.026					4.24	9	0.002
*K*_*L*_ in Rank 3	10	2.23	1.37	0.95	0.39				1.89	1.13	3.12	1.25			
Result						3.69	9	0.005					−1.77	9	0.110

The maximum of *ψ*_*L*_ (actual water potential at predawn) was inversely proportional to the DBH (*n* = 6, *R*^2^ = 0.80, *p* < 0.05), whereas no relation was found between the minimum *ψ*_*L*_ (actual water potential at midday) and DBH. That is, bigger trees had more water available in the rhizosphere. However, the strongest potential capacity to access soil water may not have been related to tree size at the time of measuring. According to the model relating *ψ*_*pd*_ and *ψ*_*md*_ proposed by Martinez-Vilalta et al. (2014), extreme strict anisohydry (*σ* > 1) was only found in Rank 3 in Jan and Rank 2 in Apr in 2011 (*n* = 10, *R*^2^ = 0.54 and 0.48, *p* < 0.05).

### Stomatal conductance and canopy conductance

Daytime hourly mean *g*_*s*_ (trees 17, 18 and 21) ranged from 0.011 to 0.151 m s^−1^. A linear relation between *g*_*s*_ and *PAR* was observed in all five time periods. In contrast, a linear relation between *g*_*s*_ and*VPD*, *SM*, and *WS* was only observed in Oct, Apr and Jul, Jan and Oct in 2011, respectively.

Canopy conductance varied seasonally (much higher in the wet season than in the dry season), which was in agreement with Kumagai et al. (2004) and Barradas et al. (2005). The *g*_*c*_ for the whole plantation ranged from 0.00058–0.034 mm s^−1^ in the wet season and 0.000092–0.070 mm s^−1^ in the dry season. Individually, the *g*_*c*_ of trees ranked 1–4 ranged from 0–0.00069 mm s^−1^. Stomatal conductance of Rank 1 trees was positively related to *g*_*c*_ in each of the five time periods (Table 5).

**Table 5:** Linear regression for *g*_*s*_ and *K*_*L*_. *g*_*s*_ was stomatal conductance; *K*_*L*_ was leaf-specific hydraulic; *R*^2^ was the coefficient of determination; *p* was significance. *p* < 0.1, *p* < 0.05, and *p* < 0.01 represented a significant, remarkable and very significant difference, respectively.

Time	Depentent variable	*n*	Independent variable	*R*^2^	*p*
Oct 2010	*g*_*s*_ in Rank 1	15	*g*_*c*_	0.67	<0.001
Jan 2011	*g*_*s*_ in Rank 1	15	*g*_*c*_	0.41	<0.01
Apr 2011	*g*_*s*_ in Rank 1	15	*g*_*c*_	0.38	<0.05
Jul 2011	*g*_*s*_ in Rank 1	15	*g*_*c*_	0.63	<0.001
Oct 2011	*g*_*s*_ in Rank 1	15	*g*_*c*_	0.85	<0.001

### Hydraulic conductance and canopy conductance

Daily *K*_*p*_ and *K*_*L*_ did not vary with tree size. A proportional relationship between *K*_*p*_ or *K*_*L*_ and *DBH* was observed in Oct 2011 (*n* = 6, *R*^2^ = 0.55 and 0.64, *p* < 0.1). Moreover, both the maximums and minimums of *K*_*p*_ and *K*_*L*_ occurred in Rank 2 and 3 trees.

There were significant differences in *K*_*p*_ and *K*_*L*_ for Rank 2 and 3 trees between 2010 and 2011 (Table 4). The *K*_*p*_ and *K*_*L*_ for Rank 2 trees in Oct 2010 was 70.69 and 86.57 g m^−2^ s^−1^ MPa^−1^, respectively. The *K*_*p*_ and *K*_*L*_ for Rank 3 trees in Oct 2010 was 1.92 and 2.23 g m^−2^ s^−1^ MPa^−1^, respectively. The *K*_*p*_ and *K*_*L*_ for Rank 2 trees in Oct 2011 was 44.37 and 36.40 g m^−2^ s^−1^ MPa^−1^, respectively. The *K*_*p*_ and *K*_*L*_ for Rank 3 trees in Oct 2011was 1.20 and 0.95 g m^−2^ s^−1^ MPa^−1^, respectively

There were significant seasonal differences for Rank 1 and 2 trees in the 2 years (Table 4). The mean daily *K*_*p*_ and *K*_*L*_ for Rank 1 trees in the wet season was 39.33 and 23.40 g m^−2^ s^−1^ MPa^−1^, respectively. The mean daily *K*_*p*_ and *K*_*L*_ for Rank 2 trees in the wet season was 0.032 and 0.013 g m^−2^ s^−1^ MPa^−1^, respectively. The mean daily *K*_*p*_ and *K*_*L*_ for Rank 1 trees in the dry season was 57.84 and 44.37 g m^−2^ s^−1^ MPa^−1^, respectively. The mean daily *K*_*p*_ and *K*_*L*_ for Rank 2 trees in the dry season was 0.016 and 0.012 g m^−2^ s^−1^ MPa^−1^, respectively.

In addition, Δ*E*_*L*_ was well related to Δ*g*_*s*_ and *K*_*L*_ in Oct 2010 and 2011, and related to *K*_*L*_ in Jan and Jul 2011, respectively (Table 3). Daily Δ*E*_*L*_, Δ*g*_*s*_ and *K*_*L*_ ranged from 0.65–10.02 g m^−2^ d^−1^, 0.01–0.20 m s^−1^, and 0.0082–0.051 g m^−2^ s^−1^ Mpa^−1^, respectively.

## Discussion

### Scaling up water use from the individual to the stand

*F*_*s*_ showed stronger relation with the ranks than *J*_*s*_ and *E*_*L*_. There are fewer studies comparing this parameter, but more interspecies studies comparing daily mean *J*_*s*_. Horna et al. (2011) compared *J*_*s*_ of seven species with no size classes in July and December between 2007 and 2008, but found no significant differences. Under a common setting criterion (i.e., depth of the xylem, azimuth), *J*_*s*_ showed high interspecific differences (Ewers et al., 2002; O’Brien, Oberbauer & Clark, 2004; Daley & Phillips, 2006; Horna et al., 2011). Even within species, *J*_*s*_ may vary with tree social position (Ambrose et al., 2010), cultural type (Kunert et al., 2012), local condition (Granier et al., 1990; Motzer et al., 2005), age (Oguntunde & Oguntuase, 2007), and tree-size parameters (Loustau et al., 1996; Ewers et al., 2002; Kume et al., 2010). Therefore, future research should sample a large number of trees to improve the accuracy (Oishi, Oren & Stoy, 2008). Many studies have provided evidence of the mechanism of this relation. Some studies suggested that variability of *J*_*s*_ is related to changes in a single environmental factor (e.g., Granier et al., 1990), while others support the influence of multiple environmental factors (Bovard et al., 2005; Daley & Phillips, 2006). Many studies that used *F*_*s*_ instead of *J*_*s*_ might have concluded different sap flow measurements, which also likely amplified individual tree differences (Roberts, Vertessy & Grayson, 2001; Tausend, Meinzer & Goldstein, 2000; Motzer et al., 2005; Yunusa et al., 2010; Zeppel et al., 2010). We found that *F*_*s*_ showed “the larger the tree, the greater the transpiration” more effectively than *J*_*s*_.*E*_*L*_ takes into account the importance of leaf area. Some studies have found that *E*_*L*_ is an important determinant of tree water use (Myers et al., 1996; Radersma, Ong & Coe, 2006). In our study, clear differences in *E*_*L*_ were also found between ranks, i.e., the leaf-area of Rank 1 was 1.48 times that of Rank 3, but transpired 51.6% more water in Jul 2011.

The seasonal decline in water use with leaf area has been shown in many studies. This has been explained as preventing canopy desiccation (Dye, 1996; Farrington et al., 1994; Hutley, O’Grady & Eamus, 2000). In this study, tree water use only accounted for 9.9% and 13.9% of rainfall in the 2010 and 2011 wet seasons, respectively, and 48.4% and 31.2% of rainfall in the 2010 and 2011 dry seasons, respectively. However, we only showed a decline in tree water use in the dry season after ignoring leaf growth. It showed the effects of external environmental factors more effectively than current approaches. Llorens et al. (2010) also showed that transpiration decreased significantly during the drier summers of 1998 and 2000, compared with the wetter summer of 1997.

Many studies have reported that *SM* determines transpiration (Jiao et al., 2015; Gazal et al., 2006; Chang et al., 2014; Zhao & Liu, 2010). Some studies have observed a decline in transpiration with decreasing *SM*. Gartner et al. (2009) found that birch and Norway spruce trees reduced their transpiration in response to drought. In this study, tree water use and canopy transpiration were significantly affected by *SM* at all daily and monthly scales. The decreasing transpiration could be partly attributed to the decrease in *SM* in the 2011 dry season, when rainfall decreased by 727.2 mm, compared with the 2010 wet season, although there were no *SM* data for 2010. Other studies have shown that rainfall influences tree transpiration when soil water in the upper profile is insufficient (O’Grady, Eamus & Hutley, 1999). Even in the afternoon, tree transpiration may vary before and after a rain event (Wang et al., 2017). Nevertheless, this effect did not show a simple proportional relation. Huang & Zhang (2016) reported that 0–5-mm precipitation increased transpiration, while >5mm precipitation decreased transpiration in two xerophytic shrubs. Conversely, water use was weakly related to rainfall, indicating that the trees strongly depended on groundwater (Morris, Mann & Collopy, 1998). The inconsistent effect of rainfall and *SM* was possibly related to root depth. Zhao & Liu (2010) showed that soil water content at 10–20-cm depth depended significantly on rainfall. It is likely that plants depend on water uptake up to 50 cm soil depth (VanSplunder et al., 1996; Lagergren & Lindroth, 2002). Our *SM* measurements at 30–40-cm depth determined the decrease in *S. superba* transpiration in the dry year (2011), which was possibly attributable to the combined effects of *SM* and rainfall. This was also shown in the decrease of 16.3% in *E*_*s*_ between the two years, which was lower than 33.8% of precipitation.

The total water use of *S. superba* in 2010 and 2011 was approximately 14.6% and 18.5% of the average annual rainfall, respectively. The low level of water consumption was possible attributed to its growth near over-mature stage. From studies of broad-leaved plantations in south China, the total annual mean daily *E*_*s*_ of 0.79 mm d^−1^ of *S. superba* (30–35a) was higher than the 0.59 mm d^−1^ of *Acacia mangium* (19a) at the Heshan experimental station in Guangdong Province (22textdegree40′N, 112°54′E, 226 m alt), and lower than the 1.01 mm d^−1^ of *Eucalyptus urophylla* ×*E. grandis* (4–5a) at the Huangmian Forest Farm in Liuzhou, Guangxi Province (24°45.8′N, 109°53.6′E, 80 m alt), and 1.48 and 1.53 mm d^−1^ of *E. urophylla* at Hetou (21°05′N, 109°54′E, 25 m alt) and Jijia (20°54′N, 109°52′E, 70 m alt), respectively, in Leizhou Peninsula, Gouangdong Province (Morris et al., 2004; Ma et al., 2008; Zhu et al., 2015). As they have similar latitude/longitude and climate background, water use of plantations poses no threat to the ground water balance, irrespective of the mix of exotic and native species, although there are differences (Myers et al., 1996).

### Stomatal and hydraulic regulation of transpiration from the individual to the stand

Regulations of *g*_*s*_ included physical factors, such as ABA, PH value, and flagellin (Small & Maxwell, 1939; Zhang, He & Assmann, 2008), and environmental factors, such as *VPD*, light intensity, air humidity, and atmospheric CO_2_ concentration (Jarvis, 1976; Aasamaa & Sõber , 2011; Monteith, 1995). Johnson et al. (2001) reported that photosynthetic photon flux density (PPFD) was most correlated with *g*_*s*_ in *Acer saccharum*. Monteith (1995) examined three phases of diurnal *g*_*s*_, corresponding to diurnal *VPD*, and found that low *VPD* increased the minimal transpiration limited by stomata (phase C), and larger values of *VPD* caused a large decline in *g*_*s*_ (phase B), while minimal medium values of *VPD* increased transpiration (phase A). Although *g*_*s*_ correlated well with *VPD* only in Oct 2011, our results followed Monteith (1995).

Extreme anisohydry was found in *S. superba*, and that seemed to be a dynamic mechanism and varied with ranks and periods. However, it is doubtful that *S. superba*, which grew in South China, showed extreme anisohydry. Martinez-Vilalta et al. (2014) found only five species showed extreme anisohydry (*σ* > 1) in 102 species. Moreover, they noted that the phenomenon may occur in phreatophytes and drought-deciduous species. In our study, *ψ*_*md*_–*ψ*_*pd*_ was approximately maintained seasonally constant but *ψ*_*pd*_ was correlated with soil water availability, which be in consistent with an isohydrodynamic behavior proposed by Franks, Drake & Froend (2007). They suggested that the behavior was linked to a combined hydraulic regulation with stomatal control and plant hydraulic conductance. We also found there were annual and seasonal coordinated regulations with stoma and hydraulic conductance on canopy transpiration (Table 3). However, it was difficult to explain the difference between Oct 2010 and Jul 2011 only linking *SM* and rainfall, because the latter had higher *SM* and rainfall. It is still suggested that a higher *SM* in the dry season (compared with Jan and Oct 2011) partially resulted in stomatal inverse control on leaf water potential (Fig. 3B), even though the inverse control was not in agreement with *ψ*_*L*_ positively controlling *g*_*s*_ reported by Comstock & Mencuccini (1998), Williams & Araujo (2002) and Ripullone et al. (2007).

Actually, instantaneous *g*_*s*_ did not effectively represent the bulk surface conductance unless it was sampled from different canopy layers. Stomatal conductance from the same canopy layer could be scaled up to calculate canopy stomatous conductance in our study (Table 5). It was merely too low values of *g*_*c*_ in our study compared to those reported for Qinhai spruce (0.3–51.3 mm s^−1^; (Chang et al., 2014)), Scots pine stands (13–28 mm s^−1^; Granier et al., 1996; Dolman et al., 1998; Sturm et al., 1998), Norway spruce stands (10–13 mm^−1^; Lu et al., 1995; Alsheimer et al., 1998; Cienciala et al., 1998), and *Fagus sylvatica* (3.3–18.5 mm^−1^; Magnani et al., 1998). However, our results concurred with the ranges reported for *Prunus armeniaca* (0.0012–0.0024 mm s^−1^; Barradas et al., 2005), and a mixed stand composed mainly of *Eucalyptus crebra* and *Callitris glaucophylla* (maximum 0.0083 mm s^−1^; Whitley et al., 2009), and a *Pinus canariensis* forest (0.0033 mm s^−1^; Kučera et al., 2017). The reported ranges of values vary greatly, not only by species (Köstner et al., 1992), age (Forrester, Collopy & Morris, 2010), temporal scales (Bernier et al., 2006), soil, root and canopy components (Morris et al., 2004), but also by different models of calculation using the same method with different parameters (e.g., Morris, Mann & Collopy, 1998; Zeppel & Eamus, 2008). In contrast, the inter-class daily conductance was smaller than that of whole trees, and the highest average conductance was 0.00069 mm s^−1^. Moreover, the maximum *g*_*c*_ always occurred in Rank 3 trees, as there were too few trees in some of the ranks, especially Rank 1. Thus, the results could not confirm that the larger trees determined the whole conductance (Martin et al., 1997).

## Conclusions

Plant-water relations of plantations have been increasingly studied, especially in south China, which has a large and growing proportion of plantation forests. The water consumption of *S. superba* trees showed no threat to the ground water balance. The annual and seasonal differences in water use were significantly affected by *SM* and rainfall. Stoma responsed to physical hydraulic factors, such as water potential, showed an isohydrodynamic behavior, and were positively linearly related with external environmental factors such as *PAR*.

##  Supplemental Information

10.7717/peerj.5164/supp-1 Data S1 Raw data All data include sap flow data, leaf water potential and stomatal conductance and environmental data.

## Additional Information and Declarations

### Data Availability

The following information was supplied regarding data availability: The raw data are provided in a  Data S1.

## References

[ref-1] Aasamaa K, Sõber A (2011). Responses of stomatal conductance to simultaneous changes in two environmental factors. Tree Physiology.

[ref-2] Alsheimer M, Köstner B, Falge E, Tenhunen JD (1998). Temporal and spatial variation in transpiration of Norway spruce stands within a forested catchment of the Fichtelgebirge, Germany. Annales des Sciences Forestières.

[ref-3] Ambrose A, Sillett S, Koch G, Van Pelt R, E Antoine M, Dawson T (2010). Effects of height on treetop transpiration and stomatal conductance in coast redwood (Sequoia sempervirens). Tree Physiology.

[ref-4] Barradas VL, Nicolás E, Torrecillas A, Alarcón JJ (2005). Transpiration and canopy conductance in young apricot (*Prunus armenica* L.) trees subjected to different PAR levels and water stress. Agricultural Water Management.

[ref-5] Bernier PY, Bartlett P, Black TA, Barr A, Kljun N, McCaughey JH (2006). Droughtconstraints on transpiration and canopy conductance in mature aspen and jackpine stands. Agricultural and Forest Meteorology.

[ref-6] Bovard BD, Curtis PS, Vogel CS, Su HB, Schmid HP (2005). Environmental controls on sap flow in a northern hardwood forest. Tree Physiology.

[ref-7] Campbell GS, Norman M (1998). An introduction to environmental biophysics.

[ref-8] Cao S, Chen L, Xu C, Liu Z (2007). Impact of three soil types on afforestation in China’s Loess Plateau: growth and survival of six tree species and their effects on soil properties. Landscape and Urban Planning.

[ref-9] Cao S, Chen L, Yu X (2009). Impact of China’s grain for green project on the landscape of vulnerable arid and semi-arid agricultural regions: a case study in northern Shaanxi Province. Journal of Applied Ecology.

[ref-10] Chang X, Zhao W, Liu H, Wei X, Liu B, He Z (2014). Qinghai spruce (Picea crassifolia) forest transpiration and canopy conductance in the upper Heihe River Basin of arid northwestern China. Agricultural and Forest Meteorology.

[ref-11] Chen L, Wang J, Wei W, Fu B, Wu D (2010). Effects of landscape restoration on soil water storage and water use in the Loess Plateau Region, China. Forest Ecology and Management.

[ref-12] Cienciala E, Kucera J, Ryan MG, Lindroth A (1998). Water flux in boreal forest during two hydrologically contrasting years; species specific regulation of canopy conductance and transpiration. Annales des Sciences Forestières.

[ref-13] Cochard H, Bréda N, Granier A (1996). Whole tree hydraulic conductance and water loss regulation in Quercus during drought: evidence for stomatal control of embolism?. Annals of Forest Science.

[ref-14] Comstock J, Mencuccini M (1998). Control of stomatal conductance by leaf water potential in Hymenoclea salsola (T. & G.), a desert subshrub. Plant, Cell and Environment.

[ref-15] Daley MJ, Phillips NG (2006). Interspecific variation in nighttime transpiration and stomatal conductance in a mixed New England deciduous forest. Tree Physiology.

[ref-16] Delzon S, Sartore M, Burlett R, Dewar R, Loustau D (2004). Hydraulic responses to height growth in maritime pine trees. Plant, Cell and Environment.

[ref-17] Dolman AJ, Moors EJ, Elbers JA, Snijders W (1998). Evaporation and surface conductance of three temperate forests in the Netherlands. Annales des Sciences Forestières.

[ref-18] Dye PJ (1996). Climate, forests and streamflow relationships in South African afforested catchments. Commonwealth Forestry Review.

[ref-19] Ewers BE, Mackay DS, Gower ST, Ahl DE, Burrows SN, Samanta SS (2002). Tree species effects on stand transpiration in northern Wisconsin. Water Resources Research.

[ref-20] Farley KA, Jobbagy EG, Jackson RB (2005). Effects of afforestation on water yield: a global synthesis with implications for policy. Global Change Biology.

[ref-21] Farrington P, Bartle GA, Watson GD, Salama RB (1994). Long-term transpiration in two eucalypt species in a native woodland estimated by the heat-pulse technique. Australian Journal of Ecology.

[ref-22] Food and Agriculature Organization of the United Nations (FAO) (2010). Global Forest Resources Assessment. http://www.fao.org/docrep/013/i1757e/i1757e.pdf.

[ref-23] Forest Stewardship Council (FSC) (2012). Strategic review on the future of forest plantations.

[ref-24] Forrester DI, Collopy JJ, Morris JD (2010). Transpiration along an age series of Eucalyptus globulus plantations in southeastern Australia. Forest Ecology and Management.

[ref-25] Franks PJ, Drake PL, Froend RH (2007). Anisohydric but isohydrodynamic: seasonally constant plant water potential gradient explained by a stomatal control mechanism incorporating variable plant hydraulic conductance. Plant Cell and Environment.

[ref-26] Gartner K, Nadezhdina N, Englisch M, Cermak J, Leitge E (2009). Sap flow of birchand Norway spruce during the European heat and drought in summer 2003. Forest Ecology and Management.

[ref-27] Gazal RM, Scott RL, Goodrich DC, Williams DG (2006). Controls on transpira-tion in a semiarid riparian cottonwood forest. Agricultural and Forest Meteorology.

[ref-28] Ge S, Zhou G, Zhang Z, Wei X, McNulty SG, Vose JM (2006). Potential water yield reduction due to forestation across China. Journal of Hydrology.

[ref-29] Granier A (1987). Evaluation of transpiration in a Douglas-fir stand by means of sap flow measurements. Tree Physiololgy.

[ref-30] Granier A, Biron P, Breda N, Pontailler JY, Saugier B (1996). Transpiration of trees and forest stands: short and long-term monitoring using sapflow methods. Global Change Biology.

[ref-31] Granier A, Biron B, Lemoine D (2000a). Water balance, transpiration and canopyconductance in two beech stands. Agricultural and Forest Meteorology.

[ref-32] Granier A, Bobay V, Gash JHC, Gelpe J, Saugier B, Shuttleworth WJ (1990). Vapour flux density and transpiration rate comparisons in a stand of Maritime pine (Pinus pinaster Ait.) in Les Landes forest. Agricultural and Forest Meteorology.

[ref-33] Guangzhou Bureau of Statistics (2010). Yearbook of Guangzhou Bureau of Statistics 2010.

[ref-34] Guangzhou Bureau of Statistics (2011). Yearbook of Guangzhou Bureau of Statistics 2011.

[ref-35] Harris PP, Huntingford C, Cox P, Gash JHC, Malhi Y (2004). Effect of soil moisture on canopy conductance of Amazonian rainforest. Agricultrual and Forest Meteorology.

[ref-36] Horna V, Schuldt B, Brix S, Leuschner C (2011). Environment and tree size controlling stem sap flux in a perhumid tropical forest of Central Sulawesi, Indonesia. Annals of Forest Science.

[ref-37] Hu XS, Tao H, Son P, Fan HL, Hong W, Wu CZ (2007). Comparison study on community structure features of the natural forest and the plantation of Schima superba. Journal of Fujian Forestry Science & Technology.

[ref-38] Huang L, Zhang Z (2016). Effect of rainfall pulses on plant growth and transpiration of two xerophytic shrubs in a revegetated desert area: Tengger Desert, China. CATENA.

[ref-39] Hutley LB, O’Grady AP, Eamus D (2000). Daily and seasonal patterns of evapotranspiration from eucalypt open-forest savanna of tropical northern Australia. Functional Ecology.

[ref-40] Jarvis PG (1976). The interpretation of the variations in leaf water potential and stomatal conductance found in canopies in the field. Philosophical Transactions of the Royal Society B: Biological Sciences.

[ref-41] Jian S, Zhao C, Fang S, Yu K (2015). Effects of different vegetation restoration on soil water storage and water balance in the Chinese Loess Plateau. Agricultural and Forest Meteorology.

[ref-42] Jiao L, Nan L, Ge S, Eric JW, Bojie F (2015). Biophysical controls on canopy transpiration in a black locust (Robinia pseudoacacia) plantation on the semi-arid Loess Plateau, China. Ecohydrology.

[ref-43] Johnson TB, Augé RM, Green CD, Stodola AJW, Olinick JB, Saxton AM (2001). Correlations of stomatal conductance with hydraulic, chemical and environmental variables in five urban tree species. Scientia Horticulturae.

[ref-44] Köstner BMM, Schulze ED, Kelliher FM, Hollinger DY, Byers JN, Hunt JE, McSeveny TM, Meserth R, Weir PL (1992). Transpiration and canopy conductance in a pristine broad-leaved forest of nothofagus: an analysis of xylem sap flow and eddy correlation measurements. Oecologia.

[ref-45] Kučera J, Brito P, Jimenez M, Urban J (2017). Direct Penman—Monteith parameterization for estimating stomatal conductance and modeling sap flow. Trees.

[ref-46] Kumagai TO, Saitoh TM, Sato Y, Morooka T, Manfroi OJ, Kuraji K, Suzuki M (2004). Transpiration, canopy conductance and the decoupling coefficient of a lowland mixed dipterocarp forest in Sarawak, Borneo: dry spell effects. Journal of Hydrology.

[ref-47] Kume T, Tsuruta K Fau-Komatsu H, Komatsu H Fau-Kumagai To, Kumagai T Fau-Higashi N, Higashi N Fau-Shinohara Y, Shinohara Y Fau-Otsuki K, Otsuki K (2010). Effects of sample size on sap flux-based stand-scale transpiration estimates. Tree Physiology.

[ref-48] Kunert N, Schwendenmann L, Potvin C, Hölscher D (2012). Tree diversity enhances tree transpiration in a Panamanian forest plantation. Journal of Applied Ecology.

[ref-49] Lagergren F, Lindroth A (2002). Transpiration response to soil moisture in pine and spruce trees in Sweden. Agricultural and Forest Meteorology.

[ref-50] Lane PNJ, Morris J, Zhang N, Zhou G, Zhou G, Xu D (2004). Water balance of tropical eucalypt plantations in south-eastern China. Agricultural & Forest Meteorology.

[ref-51] Licata JA, Gyenge JE, Fernández ME, Schlichter TM, Bond BJ (2008). Increased water use by ponderosa pine plantations in northwestern Patagonia, Argentina compared with native forest vegetation. Forest Ecology and Management.

[ref-52] Little C, Lara A, McPhee J, Urrutia R (2009). Revealing the impact of forest exotic plantations on water yield in large scale watersheds in South-Central Chile. Journal of Hydrology.

[ref-53] Llorens P, Poyatos R, Latron J, Delgado J, Oliveras I, Gallart F (2010). A multiyear study of rainfall and soil water controls on Scots pine transpiration under Mediterranean mountain conditions. Hydrological Processes.

[ref-54] Loustau D, Berbigier P, Roumagnac P, Arruda-Pacheco C, David JS, Ferreira MI, Pereira JS, Tavares R (1996). Transpiration of a 64-year-old maritime pine stand in Portugal. 1. Seasonal course of water flux through maritime pine. Oecologia.

[ref-55] Lu P, Biron P, Bréda N, Granier A (1995). Water relations of adult Norway spruce (Picea abies (L) Karst) under soil drought in the Vosges mountains: water potential, stomatal conductance and transpiration. Annals of Forest Science.

[ref-56] Lu P, Yunusa I, Walker RR, Müller JW (2003). Regulation of canopy conductance and transpiration and their modeling in irrigated grapevines. Functional Plant Biology.

[ref-57] Ma L, Lu P, Zhao P, Rao XQ, Cai XA, Zeng XP (2008). Diurnal, daily, seasonal and annual patterns of sap-flux-scaled transpiration from an Acacia mangium plantation in South China. Annals of Forest Science.

[ref-58] Magnani F, Leonardi S, Tognetti R, Grace J, Borghetti M (1998). Modelling the surface conductance of a broadleaf canopy: effects of partial decoupling from the atmosphere. Plant, Cell & Environment.

[ref-59] Martin TA, Brown KJ, Cermák J, Ceulemans R, Kucera J, Meinzer FC, Rombold JS, Sprugel DG, Hinckley TM (1997). Crown conductance and tree and stand transpiration in a second-growth Abies amabilis forest. Canadian Journal of Forest Research.

[ref-60] Martinez-Vilalta J, Poyatos R, Aguade D, Retana J, Mencuccini M (2014). A new look at water transport regulation in plants. New Phytologist.

[ref-61] Mei TT, Wang CK, Zhao P, Cai XA, Liu XJ, Zhang QZ (2010a). Dynamics of trunk sap flow density of schina superba. Scientia Silvae Sinicae.

[ref-62] Mei TT, Zhao P, WQ CXA, Yu MH, Zhu LW, Zou LL, Zeng XP (2010b). Effects of tree diameter at breast height and soil moisture on transpiration of *Schima superba* based on sap flow pattern and normalization. Chinese Journal of Applied and Environmental Biology.

[ref-63] Monteith JL (1995). A reinterpretation of stomatal responses to humidity. Plant, Cell and Environment.

[ref-64] Morris J, Mann L, Collopy J (1998). Transpiration and canopy conductance in a eucalypt plantation using shallow saline groundwater. Tree Physiology.

[ref-65] Morris J, Ningnan Z, Zengjiang Y, Collopy J, Daping X (2004). Water use by fast-growing Eucalyptus urophylla plantations in southern China. Tree Physiology.

[ref-66] Motzer T, Munz N, Küppers M, Schmitt D, Anhuf D (2005). Stomatal conductance, transpiration and sap flow of tropical montane rain forest trees in the southern Ecuadorian Andes. Tree Physiology.

[ref-67] Myers BJ, Theiveyanathan S, O’Brien ND, Bond WJ (1996). Growth and water use of Eucalyptus grandis and Pinus radiata plantations irrigated with effluent. Tree Physiology.

[ref-68] Nosetto M, Jobbágy E, Paruelo J (2005). Land-use change and water losses: the case of grassland afforestation across a soil textural gradient in Central Argentina. Global Change Biology.

[ref-69] O’Brien JJ, Oberbauer SF, Clark DB (2004). Whole tree xylem sap flow responses to multiple environmental variables in a wet tropical forest. Plant, Cell & Environment.

[ref-70] O’Grady AP, Eamus D, Hutley L (1999). Transpiration increases during the dry season: patterns of tree water use in Eucalypt open-forests of Northern Australia. Tree Physiology.

[ref-71] Oguntunde P, Oguntuase AM (2007). Influence of environmental factors on the sap flux density of mango trees under rain-fed cropping systems in West Africa. International Journal of Plant Production.

[ref-72] Oishi AC, Oren R, Stoy PC (2008). Estimating components of forest evapotranspiration: a footprint approach for scaling sap flux measurements. Agricultural and Forest Meteorology.

[ref-73] Radersma S, Ong CK, Coe R (2006). Water use of tree lines: importance of leaf area and micrometeorology in sub-humid Kenya. Agroforestry Systems.

[ref-74] Rascher K, Große-Stoltenberg A, Máguas C, Werner C (2011). Understory invasion by acacia longifolia alters the water balance and carbon gain of a Mediterranean pine forest. Ecosystems.

[ref-75] Ripullone F, Guerrier MR, Nole’ I, Magnani F, Borghetti M (2007). Stomatal conductance and leaf water potential responses to hydraulic conductance variation in Pinus pinaster seedlings. Trees.

[ref-76] Roberts S, Vertessy R, Grayson R (2001). Transpiration from Eucalyptus sieberi (L. Johnson) forests of different age. Forest Ecology and Management.

[ref-77] Schäfer KVR, Oren R, Tenhunen JD (2000). The effect of tree height on crown level stomatal conductance. Plant, Cell & Environment.

[ref-78] Small J, Maxwell KM (1939). PH phenomena in relation to stomatal opening. Protoplasma.

[ref-79] Sturm N, Köstner B, Hartung W, Tenhunen JD (1998). Environmental and endogenous controls on leaf—and standlevel water conductance in a Scots pine plantation. Annales des Sciences Forestières.

[ref-80] Tan ZH, Zhang YP, Song QH, Liu WJ, Deng XB, Tang JW, Deng Y, Zhou WJ, Yang LY, Yu GR, Sun XM, Liang NS (2011). Rubber plantations act as water pumps in tropical China. Geophysical Research Letters.

[ref-81] Tausend CP, Meinzer CF, Goldstein G (2000). Control of transpiration in three coffee cultivars: the role of hydraulic and crown architecture. Trees.

[ref-82] VanSplunder I, Voesenek LACJ, Coops H, DeVries XJA, Blom CWPM (1996). Morphological responses of seedlings of four species of Salicaceae to drought. Canadian Journal of Botany.

[ref-83] Wang H, Tetzlaff D, Dick JJ, Soulsby C (2017). Assessing the environmental controls on Scots pine transpiration and the implications for water partitioning in a boreal headwater catchment. Agricultural and Forest Meteorology.

[ref-84] Wang YL, GB L, Tomonori K, Kyoichi O, Yamanaka N, Du S (2010). Estimating water use of a black locust plantation by the thermal dissipation probe method in the semiarid region of Loess Plateau, China. Journal of Forest Research.

[ref-85] Whitley R, Medlyn B, Zeppel M, Macinnis-Ng C, Eamus D (2009). Comparing the Penman–Monteith equation and a modified Jarvis–Stewart model with an artificial neural network to estimate stand-scale transpiration and canopy conductance. Journal of Hydrology.

[ref-86] Williams L, Araujo FJ (2002). Correlations among predawn leaf, midday leaf, and midday stem water potential and their correlations with other measures of soil and plant water status in Vitis vinifera. Journal of the American Society for Horticultural Science.

[ref-87] Yunusa IAM, Aumann CD, Rab MA, Merrick N, Fisher PD, Eberbach PL, Eamus D (2010). Topographical and seasonal trends in transpiration by two co-occurring Eucalyptus species during two contrasting years in a low rainfall environment. Agricultural and Forest Meteorology.

[ref-88] Zeppel M, Eamus D (2008). Coordination of leaf area, sapwood area and canopy conductance leads to species convergence of tree water use in a remnant evergreen woodland. Australian Journal of Botany.

[ref-89] Zeppel M, Tissue D, Taylor D, Macinnis-Ng C, Eamus D (2010). Rates of nocturnal transpiration in two evergreen temperate wofodland species with differing water-use strategies. Tree Physiology.

[ref-90] Zhang W, He SY, Assmann SM (2008). The plant innate immunity response in stomatal guard cells invokes G-protein-dependent ion channel regulation. The Plant Journal.

[ref-91] Zhang ZZ, Zhao P, Ni GY, Zhu LW, Zhao XH, Zhao PQ, Niu JF (2014). Water use of re-vegetation pioneer tree species *Schima superba* and *Acacia mangium* in hilly land of South China. Chinese Journal of Applied Ecology.

[ref-92] Zhao W, Liu B (2010). The response of sap flow in shrubs to rainfall pulses in the desert region of China. Agricultural and Forest Meteorology.

[ref-93] Zhao XW, Zhao P, Zhu LW, Zhao XH (2016). Applying time series models to estimate time lags between sap flux and micro-meteorological factors. Écoscience.

[ref-94] Zheng X, Zhu JJ, Yan QL, Song LN (2012). Effects of land use changes on the groundwater table and the decline of Pinus sylvestris var. mongolica plantations in southern Horqin Sandy Land, Northeast China. Agricultural Water Management.

[ref-95] Zhou C, Wei X, Zhou G, Yan J, Wang X, Wang C, Liu H, Tang X, Zhang Q (2008). Impacts of a large-scale reforestation program on carbon storage dynamics in Guangdong, China. Forest Ecology and Management.

[ref-96] Zhou J, Zhao P, Zhu LW, Junfeng Niu, Zhao XH, Zhang ZZ, Sun ZW (2015). Wet-dry seasonal patterns and inter-tree variation of hydraulic conductance of *Schima superba*. Chinese Journal of Applied Ecology.

[ref-97] Zhu LW, Zhao P, Cai XA, Zeng X, Zou LL, Wang Q (2010). Characteristics of transpiration and canopy stomatal conductance of schima superba plantation and their responses to environmental factors. Journal of Tropical and Subtropical Botany.

[ref-98] Zhu LW, Zhao P, Wang Q, Ni GY, Niu JF, Zhao XH, Zhang ZZ, Zhao PQ, Gao JG, Huang YQ, Gu DX, Zhang ZF (2015). Stomatal and hydraulic conductance and water use in a eucalypt plantation in Guangxi, southern China. Agricultural and Forest Meteorology.

